# Topological DNA damage, telomere attrition and T cell senescence during chronic viral infections

**DOI:** 10.1186/s12979-019-0153-z

**Published:** 2019-06-24

**Authors:** Yingjie Ji, Xindi Dang, Lam Ngoc Thao Nguyen, Lam Nhat Nguyen, Juan Zhao, Dechao Cao, Sushant Khanal, Madison Schank, Xiao Y. Wu, Zheng D. Morrison, Yue Zou, Mohamed El Gazzar, Shunbin Ning, Ling Wang, Jonathan P. Moorman, Zhi Q. Yao

**Affiliations:** 1 Center of Excellence in Inflammation, Infectious Disease and Immunity, James H. Quillen College of Medicine, East Tennessee State University, Johnson City, TN 37614 USA; 2 Division of Infectious, Inflammatory and Immunologic Diseases, Department of Internal Medicine, Quillen College of Medicine, ETSU, Johnson City, TN 37614 USA; 3 Center of Cadre Health Care, The Fifth Medical Center of PLA General Hospital, Being, 100000 China; 4 Hepatitis (HCV/HBV/HIV) Program, James H. Quillen VA Medical Center, Department of Veterans Affairs, Johnson City, TN 37614 USA

**Keywords:** HBV, HCV, HIV, Topological DNA damage, Telomere attrition, T cell senescence

## Abstract

**Background:**

T cells play a key role in controlling viral infections; however, the underlying mechanisms regulating their functions during human viral infections remain incompletely understood. Here, we used CD4 T cells derived from individuals with chronic viral infections or healthy T cells treated with camptothecin (CPT) - a topoisomerase I (Top 1) inhibitor - as a model to investigate the role of DNA topology in reprogramming telomeric DNA damage responses (DDR) and remodeling T cell functions.

**Results:**

We demonstrated that Top 1 protein expression and enzyme activity were significantly inhibited, while the Top 1 cleavage complex (TOP1cc) was trapped in genomic DNA, in T cells derived from individuals with chronic viral (HCV, HBV, or HIV) infections. Top 1 inhibition by CPT treatment of healthy CD4 T cells caused topological DNA damage, telomere attrition, and T cell apoptosis or dysfunction via inducing Top1cc accumulation, PARP1 cleavage, and failure in DNA repair, thus recapitulating T cell dysregulation in the setting of chronic viral infections. Moreover, T cells from virally infected subjects with inhibited Top 1 activity were more vulnerable to CPT-induced topological DNA damage and cell apoptosis, indicating an important role for Top 1 in securing DNA integrity and cell survival.

**Conclusion:**

These findings provide novel insights into the molecular mechanisms for immunomodulation by chronic viral infections via disrupting DNA topology to induce telomeric DNA damage, T cell senescence, apoptosis and dysfunction. As such, restoring the impaired DNA topologic machinery may offer a new strategy for maintaining T cell function against human viral diseases.

## Introduction

T cells play a crucial role in defending the host from pathogenic infections; however, the precise mechanisms dampening their responses during chronic viral infections remain incompletely understood. In studying the role of T cell dysregulation in viral persistence in humans, we and others have previously shown that chronic viral infections can cause premature T cell aging and immune senescence, as evidenced by the overexpression of aging markers, such as p16^ink4a^, p21^cip1^, killer cell lectin-like receptor superfamily G 1 (KLRG1), and dual specific phosphatase 6 (DUSP6), the decline of aging-associated microRNA181 (miR181), and the particularly shortened telomeres, indicating excessive cell proliferative turnover or inadequate telomere maintenance during chronic viral infections [1–13]. Telomeres are repeating hexameric DNA sequences (TTAGGG) that are found at the chromosome ends in association with a complex of shelterin proteins [14]. Telomere integrity is a key feature of linear chromosomes that preserve genome stability and function. Conversely, telomere attrition is a hallmark of cell senescence that drives cell dysfunction and apoptosis [15–17].

The intertwined nature of two complementary DNA structures is a unique feature of chromosomes to ensure the storage and transmission of valuable genetic codes. However, almost all types of DNA activities, including gene replication, transcription, and recombination, lead to topological entanglements that must be resolved to ensure normal DNA transactions and subsequent cell functions [18]. In order to prevent and correct these types of topological problems, topoisomerases (enzymes that modulate the topology of DNA) bind to DNA and cut the phosphate backbone of either one or both DNA strands by creating transient breaks in the chromosomes [18]. This intermediate break allows the DNA to be untangled or unwound so as to exert genetic activities, and, at the end of these processes, the DNA backbone is resealed.

There are three main types of DNA topology: supercoiling, knotting, and catenation. Correspondingly, the human genome encodes three types of topoisomerases to resolve such DNA entanglements: type IA (Top3α and Top3β), type IB (nuclear Top1 and mitochondrial Top1), and type IIA (Top2α and Top2β) [18]. Topoisomerases cleave and rapidly reseal one (type I) or two (type II) DNA or RNA strands, generating a transient break through which topological modifications can occur [19]. This catalytic process is rather “dangerous” in that it generates an intermediate in which the topoisomerase becomes covalently linked to the 3′ (type IB) or 5′ (type IA and IIA) terminus of nucleic acids through a phosphotyrosine linkage, designated as the topoisomerase cleavage complex (TOPcc). Failure to complete this catalytic cycle results in trapping of topoisomerase on DNA termini, generating protein-linked DNA breaks (PDB) – a frequent event that causes cell death [20–23]. Notably, the insertion of viral or bacterial DNA into host chromosomes and other forms of recombination also requires the action of topoisomerases. Many drugs, such as broad-spectrum fluoroquinolone antibiotics and chemotherapy drugs, operate through disrupting or interfering with the function of bacterial or cancer cell topoisomerases to create breaks in chromosomal DNA and to lead cell apoptosis or dysfunction [24, 25]. Thus, although DNA topology is crucial for normal cell function, its disruption may lead to DNA damage response (DDR) and cell dysfunction or death.

While catalytic inhibition of topoisomerases has been widely exploited to kill bacterial or cancer cells [24, 25], the role and mechanisms of topoisomerases in reprogramming telomeric DDR and remodeling the function of T lymphocytes during chronic viral infections remain largely unknown. In this study, we explored the role of topoisomerase I (Top 1) in telomeric DDR, T cell senescence and dysfunction in a translational approach. We provide evidence showing that T cells derived from patients with chronic hepatitis C virus (HCV), hepatitis B virus (HBV), or human immunodeficiency virus (HIV) infection exhibit significantly inhibited Top 1 protein expression and enzyme activity, accompanied by Top1cc accumulation and trapping in genomic DNA. Using camptothecin (CPT, a Top 1 inhibitor)-treated primary T cells as a model, we demonstrate that disruption of telomeric DNA topology promotes T cell senescence, apoptosis, and dysfunction via enhancing DDR as a consequence of Top1cc being formed and trapped at the DNA break site. Importantly, T cells from virally infected subjects with inhibited Top 1 activity were more vulnerable to CPT-induced cell apoptosis, indicating an important role for Top 1 in maintaining DNA integrity and securing cell survival. These results suggest that immunocompromising viruses (HCV, HBV, and HIV) may disrupt this T cell DNA topological processes as a mechanism to dysregulate host immunity and establish chronic viral infection. Thus, restoring the impaired DNA topologic machinery may serve as a novel strategy to protect T cells from unwanted DNA damage and maintain immune competency.

## Materials and methods

### Subjects

The study subjects were composed of four populations: 37 chronically HCV-infected patients prior to antiviral therapy; 29 chronically HBV-infected patients on antiviral treatment with undetectable viremia (HBV-DNA); 28 latently HIV-infected patients on antiretroviral therapy (ART) with undetectable viremia (HIV-RNA); and 144 age-matched healthy subjects (HS), which were obtained from Physicians Plasma Alliance (PPA), Gray, TN, and negative for HBV, HCV and HIV infection. The characteristics of the subjects included in this study are described in Table 1.

**Table 1:** Demographic information of the study participants

Subjects	Numbers	Age (Mean)	Gender (M/F)	Viral load and other characteristics
HCV	37	31–69 (52)	29/8	17,000-9,980,000 IU/ml, 26 GT1, 6 GT2, 5 GT3
HBV	29	22–61 (49)	20/9	All on antivirals with undetectable HBV-DNA
HIV	28	25–62 (46)	21/7	All on ART with undetectable HIV-RNA
HS	144	24–58 (44)	112/32	All tested negative for HCV, HBV, and HIV

### Cell isolation and culture

Peripheral blood mononuclear cells (PBMCs) were isolated from whole blood by Ficoll (GE Heathcare, Piscataway, NJ) density centrifugation. CD4 T cells were isolated from PBMCs using the CD4 T Cell Negative Isolation Kit and a MidiMACS™ Separator (Miltenyi Biotec Inc., Auburn, CA). The isolated T cells were cultured in RPMI 1640 medium containing 10% FBS (Atlanta Biologicals, Flowery Branch, GA), 100 IU/ml penicillin and 2 mM L-glutamine (Thermo Scientific, Logan, UT) at 37 °C and 5% CO_2_ atmosphere.

### Flow cytometry

Intracellular IL-2 and IFN-γ cytokine production, DNA damage marker γH2AX expression, PKH26-label CD4 T cell proliferation, telomere length measured by florescence in-situ hybridization (Flow-FISH), and cell apoptosis assay for Av/7AAD expression were analyzed by flow cytometry, as described previously [26, 27]. The following reagents were used in the assays: PE-labelled Av, IL-2, IFN-γ, γH2AX (BD Biosciences, San Jose, CA), CD4-Alexa-647 (BioLegend, San Diego, CA), telomere probe TelC (CCCTAACCCTAACCCTAA)-FITC (0.25 μg probe/mL, PNA Bio, Newbury Park, CA).

### RNA isolation and real-time RT-PCR

Total RNA was extracted from 1 × 10^6^ CD4 T cells using PureLink RNA Mini Kit (Invitrogen, Carlsbad, CA), and cDNA was synthesized using High Capacity cDNA Reverse Transcription Kit (Applied Bio systems, Foster city, CA) per the manufacturer’s instructions. Quantitative real-time polymerase chain reaction (PCR) was performed in triplicate as described previously [27]. Gene expression was determined using the 2^-ΔΔct^ method and normalized to GAPDH levels, and is presented as fold changes. The PCR primer sequences are shown in Table 2.

**Table 2:** PCR primers used in this study

Primers	Forward	Reverse
TOP1	5′-TCCGGAACCAGTATCGAGAAGA-3′	5′-CCTCCTTTTCATTGCCTGCTC-3’
hTERT	5′- CCAAGTTCCTGCACTGGCTGA-3’	5′- TTCCCGATGCTGCCTGACC-3’
TRF1	5′-TGCTTTCAGTGGCTCTTCTG-3’	5′-ATGGAACCCAGCAACAAGAC-3’
TRF2	5′-GGTACGGGGACTTCAGACAG-3’	5′-CGCGACAGACACTGCATAAC-3’
TPP1	5′-TCACCAGATCAGCCACATTC-3’	5′-TGGAAAGACTCTCGGAGCTG-3’
POT1	5′-TTCCACTAAAGAGCAGGCAA-3’	5′-TGAAGTTCTTTAAGCCCCCA-3’
TIN2	5′-TGCTTTCAGTGGCTCTTCTG-3’	5′-TTTACCAGCAGGTGAAGCAG-3’
RAP1	5′-TCTTCTTCAGGCAAATCTGGA-3’	5′-CCTCCTCCCAGAAGCTCAA-3’
GAPDH	5′-TGCACCACCAACTGCTTAGC-3’	5′- GGCATGGACTGTGGTCATGAG-3’

### Western blotting

CD4 T cells (2 × 10^6^) purified from HCV, HBV, or HIV patients and HS were used for western blot as described previously [26, 27]. Primary and secondary antibodies included Top1, PARP1, γH2AX, TRF1, TRF2, TPP1, TIN2, RAP1, POT1, ATM, pATM, CHK2, ATR, GAPDH, β-actin, and horseradish peroxide-conjugated antibodies (Cell Signaling). Images were captured using ChemiDoc™ XRS+ System (Bio-Rad). Protein band intensity was quantitated by the Image Lab software (Bio-Rad).

### Top 1 activity assay

The activity of Top 1 was measured by Topoisomerase 1 Assay Kit (Topogen, Inc., Cal No: TG1015–1, Buena Vista, CO). Briefly, CD4 T cells were purified from patients and HS, and cell extracts were isolated from the purified cells according to the instructions of the kit. Samples were mixed with plasmid DNA substrate and reaction buffer for 30 min at 37 °C, then loaded onto a 1% agarose gel with loading dye, and electrophoresed for about 2 h at 5~10 V/cm before illuminating with a UV transilluminator. The intensity of supercoiled DNA substrate was measured by densitometry.

### Top1cc detection

Top1cc was detected using the Human Topoisomerase ICE Assay Kit (Topogen, Inc., Cal No: TG1020–1, Buena Vista, CO 81211). The DNA purification protocol was modified by combining ICE Assay Kit and PureLink™ Genomic DNA Mini Kit (Thermo Fisher Scientific, Catalog number: K182001, Waltham, MA 02451). Briefly, genomic DNA sample were isolated from cell pellets using the extraction buffers of ICE assay kit and then purified by the column of PureLink™ Genomic DNA Mini Kit. The DNA samples were loaded onto NC membranes by a vacuum pump, which was incubated with primary anti-Top1cc antibody from ICE assay kit, followed by western blotting as described above.

### Confocal microscopy

CD4 T cells were isolated and treated as described above, followed by immunofluorescence staining using a method described previously [26]. Rabbit anti-53BP1, anti-Ku70, anti-RAD51 or anti-Top1cc (Cell Signaling) and mouse TRF1 (Thermo Fisher) were used as primary antibodies and anti-rabbit IgG-Alexa Fluor 488 and anti-mouse IgG-Alexa Fluor 555 (Invitrogen) were used as secondary antibodies. The cells were washed and mounted with DAPI Fluoromount-G (SouthernBiotech, Birmingham, AL). Images were acquired with a confocal laser-scanning inverted microscope (Leica Confocal, Model TCS sp8, Germany).

### Statistics

The data were analyzed using Prism 7 software, and are presented as mean ± SEM or median with interquartile range. Comparisons between two groups were made using unpaired Student’s *t* test, or paired T test. *P*-values < 0.05, < 0.01, or < 0.001 were considered statistically significant or very significant, respectively.

## Results

### Top 1 expression and activity are inhibited in CD4 T cells from individuals with chronic viral infections

**Fig. 1:**
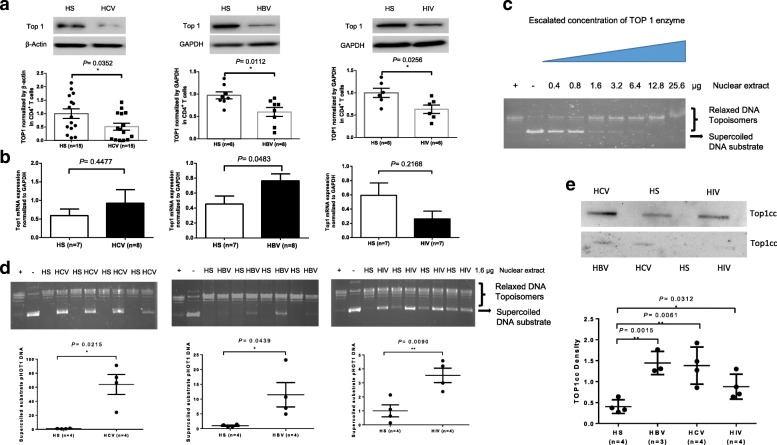
Inhibition of Top 1 expression and activity in CD4 T cells during chronic viral infections. **a** Top 1 protein expression in CD4 T cells isolated from HCV-, HBV-, and HIV-infected individuals versus HS. Representative imaging and summary data of western blot are shown. The Top 1 densitometry values were normalized to β-actin and then HS. **b** Top 1 mRNA expression, measured by real-time RT-PCR, in CD4 T cells isolated from virally infected individuals and HS. **c** Dose-dependent Top 1 enzyme activity measured by a plasmid (pHOT1)-based Top 1 Assay Kit. **d** Top 1 activity in CD4 T cells isolated from HCV-, HBV-, and HIV-infected individuals versus HS. Representative imaging and summary data of Top 1-mediated digestion of supercoiled DNA substrate (normalized to HS) are shown (n = number of subjects) to be tested. **e** Top1cc detected in genomic DNA isolated from CD4 T cells of virus-infected patients versus HS. HS, health subject; n, number of subjects

Chronic viral (HCV, HBV, HIV) infections are characterized by T cell exhaustion, senescence, and cellular dysfunction [1–13], but the underlying mechanisms remain incompletely understood. We have recently discovered that these dysfunctional, senescent T cells exhibit pronounced DNA damage and telomere erosion [26, 27]. Given the crucial role of DNA topology in securing genomic integrity and cell survival [20–23], we used a translational approach to explore the mechanisms of DNA damage and T cell dysregulation by examining the expression level of Top 1 in CD4 T cells derived from individuals with chronic viral (HCV, HBV, HIV) infection and HS. As shown in Fig. 1a, chronically HCV, HBV, or HIV-infected patients exhibited a significantly lower level of Top 1 protein expression in their CD4 T cells compared to age-matched HS, as determined by western blotting. To determine whether Top 1 inhibition occurs at the transcriptional or translational level, we measured Top 1 mRNA levels by real-time RT-PCR in CD4 T cells derived from these subjects. As shown in Fig. 1b, the mRNA levels of Top 1 in CD4 T cells isolated from these patients showed little changes compared to age-matched HS, suggesting that Top 1 inhibition during chronic viral infections primarily occurs at the translational level.

Human Top 1 is a type 1B topoisomerase that can relax (change DNA linking in step one) either positive or negative supercoiled DNA [20]. Thus, we employed a plasmid (pHOT1)-based Top 1 Assay Kit (TopoGEN, Inc.) to measure Top 1 activity in CD4 T cells derived from patients with chronic viral infection. As shown in Fig. 1c (left to right), where the Top 1-relaxed linear plasmid DNA (pHOT1) served as positive control (+), the untreated supercoiled plasmid DNA served as negative control (−), and an escalated amount of nuclear extract-treated plasmid DNA exhibited a pattern from supercoiled DNA substrate to linear DNA topoisomers in 1% agarose gel in a dose-dependent manner. Based on this result, we used 1.6 μg of nuclear extracts, which can resolve > 50% supercoiled DNA substrate, to compare the Top 1 enzyme activity in virus-infected patients versus HS. While the HS-derived nuclear extracts efficiently relaxed the supercoiled plasmid DNA, the nuclear extracts derived from HCV-, HBV- and HIV-infected patients failed to completely relax the plasmid DNA (Fig. 1d).

Top 1 is a prototypical eukaryotic enzyme that relaxes supercoiled DNA without the need for cellular energy (ATP). During the course of its normal catalytic cycle, Top 1 nicks one DNA strand and allows rotation around the intact strand, and then reseals the DNA backbone. This DNA relaxation activity plays an important role in restoring DNA topology, replication, transcription, and viral integration [18–25]. Collisions of this catalytic cycle with the Top1cc formed during DNA nicking are thought to produce further DNA damage, ultimately leading to cell death [20–23]. To determine if Top1 inhibition resulted in Top1cc buildup and trapping in their T cell chromosomes, we measured Top1cc in genomic DNA isolated from CD4 T cells of virus-infected patients and HS using a monoclonal antibody that specifically recognizes covalent Top 1-DNA complexes, but not free Top 1 or DNA, using immunoblotting [28]. As shown in Fig. 1e, a significant amount of Top1cc was detected in CD4 T cells isolated from HCV, HBV, HIV-infected patients compared to HS. These results, in conjunction with our previous findings of T cell exhaustion and senescence with poor cellular functions during chronic viral infections [1–4, 26, 27], suggest that these dysfunctional, senescent T cells from chronically virus-infected individuals have topological aberrancies, i.e., Top 1 inhibition, leading to Top1cc accumulation.

### Top 1 inhibition by CPT in healthy T cells induces telomeric DNA damage, cell apoptosis and dysfunction

**Fig. 2:**
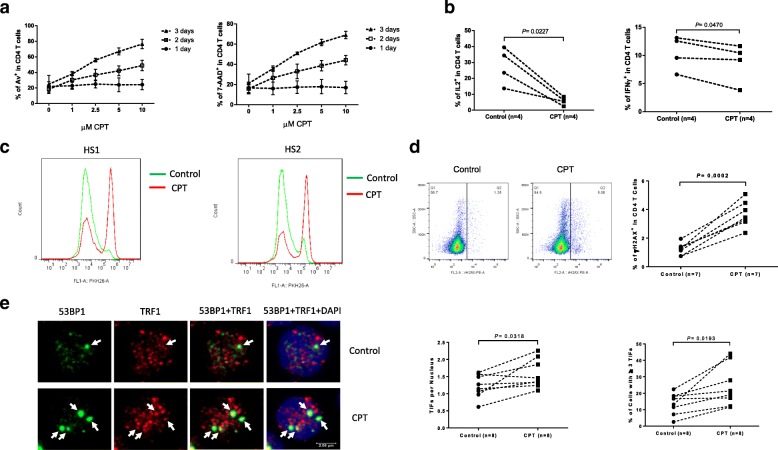
Top 1 inhibition induces telomeric DNA damage, T cell apoptosis, and T cell dysfunction. **a** Dose- and time-dependent induction of Av and 7-AAD expression in CD4 T cells treated with varying concentrations of CPT for 1, 2, and 3 days, analyzed by flow cytometry. **b** Inhibition of IL-2 and IFN-γ expression in CD4 T cells treated with or without 1 μM CPT for 3 days, followed by flow cytometry. **c** Flow cytometry of T cell proliferation as determined by PKH26 dye dilution in CD4 T cells treated with or without 1 μM CPT for 5 days. **d** γH2AX expression in CD4 T cells treated with or without 1 μM CPT for 3 days followed by flow cytometry. **e** Confocal microscopy of Telomeric DNA damage foci to assess co-localization of 53BP1 and TRF1 on telomeres, in CD4 T cells treated with or without 1 μM CPT for 2 days. Representative images and summary data for TIFs per nucleus as well as percentage of cells with > 3 TIFs are shown

Top 1 is targeted by a class of widely utilized anticancer drugs [18, 19]. CPT intercalates into DNA at the Top 1 catalytic site, inhibiting the reseal step and shifting the equilibrium toward Top1cc accumulation [29]. To explore the role of Top 1 inhibition in DNA damage and T cell dysregulation, we employed CPT-treated primary CD4 T cells as a model. Since Top1cc can be selectively trapped by CPT, which binds at the Top 1-DNA interface, leading to potent transcription- and replication-blocking DNA lesions causing cytotoxicity [29]. To this end, CD4 T cells were isolated from HS and exposed to varying concentrations of CPT (0, 1, 2.5, 5, 10 μM) for different time points (24, 48, 72 h), followed by the measurement of cell apoptotic death by flow cytometry. As shown in Fig. 2a, CPT-treated healthy T cells exhibited a dose- and time-dependent increases in Annexin V (Av, left panel) and 7-Aminoactinomycin D (7AAD, right panel) levels compared to cells treated with dimethyl sulfoxide (DMSO) control. We also measured T cell functions by flow cytometric analysis of intracellular cytokine production and cell proliferation following T cell receptor (TCR) stimulation and with or without CPT treatment. As shown in Fig. 2b, intracellular IL-2 and IFN-γ cytokine productions were significantly inhibited in human T cells exposed to CPT compared to DMSO control in the presence of anti-CD3/CD28 (1 μg/ml) stimulation for 3 days. Moreover, a significantly inhibited T cell proliferation, as evidenced by the delayed PKH26 dye dilution, was observed in human T cells exposed to CPT compared to control, in the presence of TCR stimulation for 5 days (Fig. 2c).

To determine whether CPT-induced DNA damage might be a primary reason for T cell dysfunction, we treated CD4 T cells with CPT or control for 3 days, followed by measuring the phosphorylated histone H2AX (γH2AX), a marker for DNA damage. As shown in Fig. 2d, a significant γH2AX upregulation was detected in CPT-treated CD4 T cells compared to the control, as determined by flow cytometry. Notably, these findings (CPT-induced T cell apoptosis, cytokine or proliferation inhibition via DDR in vitro) recapitulate the T cell dysregulation due to exhaustion and senescence we and others have observed in chronic viral infections in vivo [1–13, 26, 27]. Therefore, CPT-treated T cells could serve as a model to study the role and mechanisms of Top 1-mediated DNA topological aberrancies in T cell dysregulation.

Human T cell telomeres shorten at a rate of 50~100 base pairs per cell division, acting as a molecular clock to control the replicative capacity before entering cell cycle arrest, senescence, or apoptosis [17]. Since mammalian telomeres consist of triple guanine repeats (TTAGGG) that are very sensitive to oxidative DNA damage [30, 31], we speculated that CPT-induced DNA damage may extend to the telomeres that we have shown to have telomeric DNA damage in T cells derived from chronically virus-infected individuals [26, 27]. Notably, following genotoxic insult, 53BP1 is recruited to the DNA damage site on chromosomes as well as telomeres and acts as a docking station for other mediator or adaptor proteins to form microscopically visible nuclear foci (DNA damage foci) [32, 33]. A single 53BP1-formed focus represents hundreds to thousands of 53BP1 proteins that are recruited around at least one DNA strand break [32]. Thus, identifying damaged telomere induced foci (TIF) is typically regarded as a hallmark of telomeric DNA damage [33]. To determine telomeric DNA damage in CPT-treated T cells, we compared the number of TIFs per nucleus and the percentages of cells with > 3 TIFs, by examining the co-localization of 53BP1/TRF1 (telomeric repeat-binding factor 1) using confocal microscopy. As shown in Fig. 2e, the numbers of TIF per nucleus were significantly higher in CD4 T cells exposed to CPT vs. DMSO-treated control. The percentage of T cells with > 3 TIFs was also higher in CPT-treated cells compared to the control. Taken together, these results suggest that CPT-induced topological DNA damage may cause cell apoptosis and T cell dysfunction, thus emphasizing the role of telomere integrity in securing T cell survival and providing a robust model to study Top 1-mediated telomere topological DNA damage and repair machinery.

### CPT-induced DNA damage involves Top1cc accumulation, PARP-1 cleavage, and transcription-dependent, ubiquitin-mediated top 1 proteolysis

**Fig. 3:**
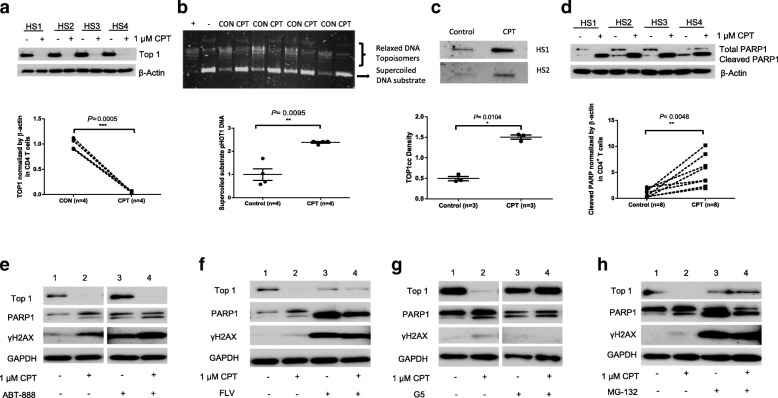
Top 1 inhibition, Top1cc and PARP1 induction, and transcription-dependent ubiquitination of Top 1 in CPT-treated T cells. **a** Western blot analysis of Top 1 inhibition in CD4 T cells treated with or without 1 μM CPT for 3 days. **b** Top 1 enzyme activity measured in CD4 T cells exposed to CPT and control for 3 days. **c** Top1cc detected in genomic DNA from CD4 T cells treated with or without CPT (50 μM for 45 min). **d** PARP1 induction in CD4 T cells with or without CPT treatment. **e** Western blot analysis of Top 1, PARP1, and γH2AX levels in CD4 T cells with or without CPT treatment in the presence or absence of APRP-1 inhibitor (ABT-888). **f** Western blot analysis of Top 1, PARP1, and γH2AX levels in CD4 T cells with or without CPT treatment in the presence or absence of transcription inhibitor (FLV). **g** Western blot analysis of Top 1, PARP1, and γH2AX levels in CD4 T cells with or without CPT treatment in the presence or absence of ubiquitin isopeptidase inhibitor (G5). **h** Western blot analysis of Top 1, PARP1, and γH2AX levels in CD4 T cells with or without CPT treatment in the presence or absence of proteasomal inhibitor (MG-132)

Physiologically, Top 1 relaxes intertwined DNA by producing transient Top1cc during DNA transcription. After DNA relaxation, Top1cc reverses rapidly and Top 1 is released as the DNA re-ligates. However, Top1cc can be trapped under pathological conditions, and a consequence of transcription-blocking Top1cc is the production of Top1-lined PDBs [29]. To determine whether CPT-induced T cell inhibition is primarily due to Top 1 suppression, we examined Top 1 protein expression in CD4 T cells treated with CPT by western blot. As shown in Fig. 3a, Top 1 protein expression was significantly inhibited in CD4 T cells exposed to CPT. In addition, Top 1 enzyme activity was also inhibited in CD4 T cells treated by CPT compared to the control (Fig. 3b). To determine whether Top1cc accumulated during DNA nicking in T cells exposed to CPT, we treated primary CD4 T cells with a high dose of CPT (50 μM) for a short period of time (45 min), followed by genomic DNA isolation and immunoblotting [28]. Of note, Top1cc was found to be trapped at genomic DNA in CPT-treated T cells compared to the DMSO control (Fig. 3c). These observations recapitulated the Top 1 inhibition seen in patients with chronic viral infections (Fig. 1).

Trapped Top1cc can cause DNA strand breaks [34, 35], and removal of these breaks depends on the tyrosyl-DNA phosphodiesterase-1 (TDP1) excision pathway [22, 36–40]. Notably, Top1cc excision by TDP1 requires Poly ADP-Ribose Polymerase 1 (PARP1), an enzyme that catalyzes the transfer of ADP-ribose onto target proteins and plays an important role in cell apoptosis, DNA damage repair and chromosomal stability [40–42]. PARP1 binds to TDP1, leading to TDP1 stabilization and its recruitment at the sites of Top1cc-induced PDB to initiate the repairing process. To determine whether CPT-induced DNA damage in T cells involves PARP1 cleavage, we measured PARP1 in CPT-treated CD4 T cells. As shown in Fig. 3d, while the total PARP1 level was reduced, the cleaved form of PARP1 was significantly induced by the CPT treatment, which correlated with DNA damage and cell apoptosis.

Since PARP1 binding to TDP1 is crucial for Top1cc removal [40–42], we then assessed whether inhibition of PARP1 would increase DNA damage in CPT-treated T cells. As shown in Fig. 3e, compared to the DMSO-treated control (1), CPT treatment (2) significantly inhibited Top 1 level but increased PARP1 cleavage and γH2AX expression. Whereas the PARP1 inhibitor ABT-888 (also known by its trade name, Veliparib, as a chemotherapeutic) [43] did not block (but feedback increased) Top 1 expression, it augmented the levels of PARP1 and γH2AX in DMSO (3) as well as CPT-treated (4) CD4 T cells (4), indicating that PARP1 is a down-stream molecule acting together with CPT in the Top 1 signaling pathway.

Collisions of transcription complexes with CPT-induced Top1cc are thought to produce DNA damage and cell apoptosis. Consistent with this model, we speculated that inhibition of transcription would diminish the DNA damage of Top 1 poisons. To test whether CPT-induced, Top 1-mediated DNA damage is transcription-dependent, we examined Top 1, PARP1, and γH2AX levels in CD4 T cells treated with CPT in the presence or absence of the transcription inhibitor flavopiridol (FLV). As shown in Fig. 3f, compared to DMSO-treated control (1), CPT treatment (2) inhibited Top 1, but increased PARP1 cleavage and γH2AX expression. As expected, while FLV inhibited Top 1 and significantly boosted PARP1 cleavage and γH2AX expressions (3), inhibition of transcription prevented CPT-induced Top 1 suppression and further induction of PARP1 or γH2AX levels in CD4 T cells (4). These results indicate that CPT induces a transcription-dependent DDR in human T lymphocytes.

As we have shown above, Top 1 inhibition primarily occurs at the protein level (Fig. 1). Indeed, Top1cc-mediated transcription blockade triggers Top 1 degradation by the ubiquitin/proteasome system [44]. Thus, we further examined whether the protein degradation machinery could contribute to the observed Top 1 protein inhibition and occurrence of transcription-dependent DNA damage. As shown in Fig. 3g, compared to the DMSO-treated control (1), CPT treatment inhibited Top 1 level, but induced PARP1 and γH2AX levels (2). Intriguingly, inhibition of ubiquitin by isopeptidase inhibitor (G5), which causes depletion of free nuclear ubiquitin [45], prevented Top 1 degradation in CPT-treated T cells (4). We then assessed whether inhibiting the ubiquitin system could prevent DDR signaling. Notably, the ubiquitin inhibitor G5 could induce PARP1 cleavage and γH2AX expression in primary CD4 T cells (3); however, G5 treatment prevented further induction of PARP1 and γH2AX levels in CPT-exposed cells (4). Similarly, inhibition of proteolysis by a proteasome inhibitor (MG132), which prevented CPT-induced Top 1 degradation, also prevented CPT-induced accumulation of PARP1 and γH2AX (Fig. 3h), suggesting that topological DNA damage depends upon Top 1 ubiquitination and proteasome degradation. Taken together, these results indicate that the CPT-induced topological DNA damage involves the transcription-dependent, ubiquitin-mediated Top 1 proteolysis.

### CPT-induced top 1 inhibition induces telomere attrition via disruption of telomerase activity and shelterin protection

**Fig. 4:**
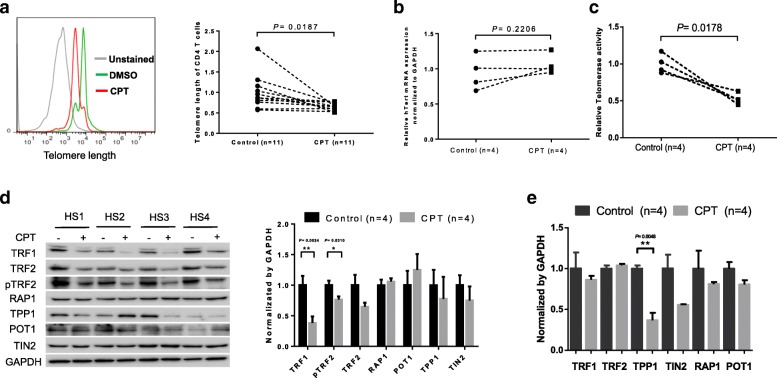
Top 1 inhibition induces telomere erosion via suppression of telomerase activity and shelterin proteins. **a** Telomere length, measured by Flow-FISH, in CD4 T cells with or without CPT treatment for 5 days. **b** Telomerase hTERT expression, measured by real-time RT-PCR, in CD4 T cells treated with or without CPT. **c** Telomerase activity, measured by TRAP assay, in CD4 T cells treated with or without CPT. **d** Shelterin proteins, measured by western blot, in CD4 T cells treated with or without CPT. **e** Shelterin mRNA, measured by real-time RT-PCR, in CD4 T cells treated with or without CPT

We and others have recently shown that T cell exhaustion and senescence during chronic viral infections are associated with accelerated telomere erosion, a hallmark of cell aging or immune senescence [4–7, 26, 27]. To determine whether CPT-treated T cells mirror the telomere loss seen in patients with viral infections, we measured the telomere length in CPT-treated T cells by Flow-FISH. As shown in Fig. 4a, significant telomere attrition was observed in CD4 T cells exposed to CPT compared to the DMSO-treated control. Notably, telomeres are replenished by telomerase that comprises telomerase RNA (TR) and human telomerase reverse transcriptase (hTERT) - the catalytic unit of telomerase [46]. Telomerase regulation has been studied extensively in malignant cells, and recently, hTERT measurement has also been employed to study T cells that have a high rate of turnover, especially during pathogenic infections [47, 48]. We have recently measured telomerase expression and activities in CD4 T cells derived from patients with chronic HCV and/or HIV infection, and found that hTERT expression remained unchanged, but its activity was significantly suppressed and failed to be recruited onto telomeres to maintain telomere integrity (unpublished observations). Here, we further measured hTERT expression by real-time RT-PCR and telomerase activities by TRAP assay in CPT-treated CD4 T cells. As shown in Fig. 4b and c, CPT-treated T cells exhibited an unchanged hTERT expression but remarkably inhibited telomerase activity when compared to the DMSO-treated control, which recapitulates the observations in T cells from patients with HCV and HIV infection.

It is well-known that telomeres are protected by shelterin complexes. To explore the mechanisms of telomere erosion in CPT-treated T cells, we examined the integrity of the telomeric shelterin complex, which comprises six telomere-associated proteins, including telomeric repeat-binding factors 1 and 2 (TRF1 and TRF2), repressor/activator protein 1 (RAP1), telomere protection protein 1 (TPP1), protection of telomere 1 (POT1), and TRF1-interacting nuclear protein 2 (TIN2), where they act together in shaping and safeguarding telomeres from erosion [49, 50]. As shown in Fig. 4d, TRF1 and pTRF2 levels were significantly lower than control levels. TPP1 and TIN2 levels were slightly downregulated, whereas RAP1 and POT1 remained unchanged or slightly upregulated, in CD4 T cells after the CPT treatment. However, the mRNA expression of these shelterin proteins remained essentially unchanged, except TPP1, by this treatment (Fig. 4e). These results recapitulate the data we observed in CD4 T cells derived from HCV and/or HIV-infected patients versus HS [27] (unpublished observations). Since the primary functions of TRF1/TRF2/TPP1/TIN2 sub-complexes are to protect telomeres from unwanted DNA damage and to recruit telomerase to telomeres, their inhibition would lead to telomere uncapping or deprotection, and a telomerase that is unable to access or function at telomeres.

### CPT-mediated top 1 inhibition and DDR involves dynamic activation and depletion of DNA repair kinases

**Fig. 5:**
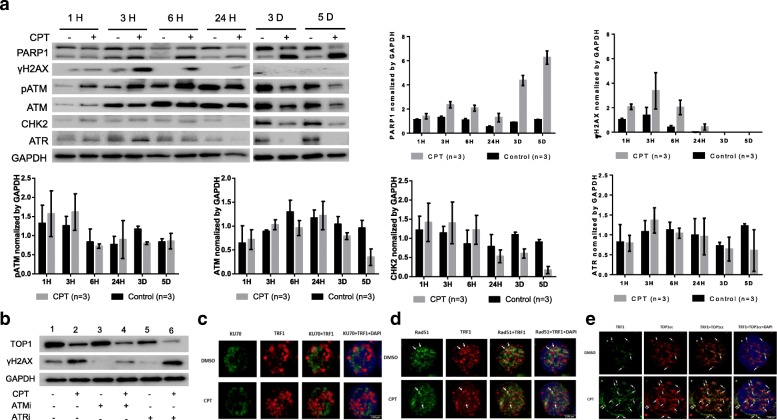
Top 1-mediated DDR involves dynamic activation and deficiency of DNA repair kinases. **a** Western blot analysis of kinetic alterations in PARP1, γH2AX, pATM, ATM, CHK2, and ATR levels in CD4 T cells treated with or without CPT for different time points. Representative images and summary data from 3 independent experiments are shown. **b** Western blot of Top 1 and γH2AX expression in CD4 T cells treated with or without CPT in the presence or absence of ATMi or ATRi. **c** Representative images from confocal microscopy examination of co-localization of Ku70 and TRF1 in CD4 T cells treated with CPT or DMSO for 3 days. **d** Representative images of co-localization of RAD51 and TRF1 in CD4 T cells treated with CPT or DMSO for 3 days. **e** Representative images of co-localization of Top1cc and TRF1 in CD4 T cells treated with CPT or DMSO for 3 h

Accumulation of DNA damage activates the protein kinase ataxia telangiectasia mutated (ATM), an enzyme critically involved in repairing DNA damage for cell survival [51, 52]. ATM, along with ataxia telangiectasia Rad3-related (ATR), is the pinnacle kinase of the DNA repair signaling cascade, which is important for DNA reprogramming and T cell rearrangement [53, 54]. To investigate the involvement of DNA repair enzymes in CPT-mediated Top 1 inhibition and topological DDR, we examined the kinetic expressions of ATM and ATR, in conjunction with DNA damage and repair markers, γH2AX and PARP1, in CD4 T cells following CPT treatment for different time points (1 h, 3 h, 6 h, 1 d, 3 d, 5 d). As shown in Fig. 5a, an increase in PARP1 cleavage was seen in CPT-treated T cells at all time points. Also, γH2AX was increased in the early phase (1~6 h) of CPT treatment; however, it decreased and eventually could not be detected in CD4 T cells at later times after CPT treatment (3~5 d), which is consistent with a previous report indicating that CPT treatment promotes DDR and activates DNA protein kinase c (DNA-PKc), and that ATM enhances phosphorylation of H2AX at an early phase and then triggers the ubiquitination cascade and proteasome degradation of γH2AX at double strand break (DSB) sites at later stages [55]. Indeed, ATM and pATM were increased in response to DDR in the early phase (1~6 h) and then gradually became deficient in the later phase of CPT treatment (3~5 d). Correspondingly, the downstream checkpoint kinase 2 (CHK2) was also upregulated in the early phase and became progressively depleted in the late phase of CPT treatment. Likewise, ATR exhibited the same pattern as ATM in response to the acute and persistent DDR induced by CPT treatment. In conjunction with our recent findings of ATM insufficiency in CD4 T cells in chronic HCV and HIV infection [26] (unpublished observations), these data indicate that CPT-induced, Top1-mediated topological DDR involves a dynamic activation and depletion of DNA repair kinases.

Next, we studied the role of these DNA repair kinases (ATM/ATR) in T cell topological DDR to gain insight into their functional relevance. To identify which of these kinases induces γH2AX in topological DDR, we assessed whether γH2AX phosphorylation is prevented by the specific inhibitors of these kinases in CPT-induced TOP 1 inhibition. Since γH2AX induction was primarily observed in the early phase (3~6 h) of CPT treatment (Fig. 5a), we chose to measure its level in CD4 T cells after 3 h of CPT treatment. As shown in Fig. 5b, CPT treatment inhibited Top 1 expression. Correspondingly, the expression of γH2AX was increased by the CPT-induced topological DDR (2 vs.1). ATMi (KU60019) did not affect TOP 1 level, but significantly reduced γH2AX level (3 vs.1). Interestingly, ATMi reduced the CPT-mediated γH2AX induction (4 vs. 2), suggesting that ATMi inhibits DDR. In addition, while ATRi did not affected TOP 1 level as well, it reduced the level of γH2AX (5 vs.1), which is consistent with the ATMi treatment, suggesting that these kinase inhibitors inhibit DDR. However, ATRi treatment further reduced the CPT-mediated Top 1 downregulation and, concurrently, further induced the γH2AX expression (6 vs.2). We observed similar results in repeated experiments, and these are consistent with previous reports [55], suggesting that ATM, but not ATR, is implicated in the induction of γH2AX in CPT-treated cells.

### CPT induces Top1cc accumulation to prevent DNA repair molecule recruitment to the DNA damage sites

We have recently found that the non-homologous end joining (NHEJ) pathway, rather than the homologous recombination (HR) pathway of DNA repair, participates in the ATM-mediated telomeric DNA damage repair (unpublished data). However, how DSBs accumulate in cells despite the presence of DNA repair proteins remains unknown. It is well-known that Ku70 and Ku80 make up the Ku heterodimer, which binds to DNA-DSB ends and is required for the NHEJ pathway of DNA repair. It is also required for V(D)J recombination, which utilizes the NHEJ pathway to promote antigen diversity in the mammalian immune system. In addition to its role in NHEJ, Ku is involved in telomere length maintenance [56]. It has been reported that mutant mice with deficient Ku70 exhibit premature aging [57], suggesting that Ku70 plays an important role in longevity assurance and that the reduced ability to repair DNA-DSB causes early aging. To determine the role of the NHEJ pathway in repairing CPT-induced telomeric DNA damage, we measured the Ku70-dependent, damaged telomere induced foci (TIF) in CD4 T cells exposed to CPT or DMSO for 6 or 72 h using confocal microscopy. Surprisingly, we could not identify any Ku70/TRF1 co-localization in cells treated with CPT or DMSO control (Fig. 5c), suggesting that Ku 70 failed to be recruited to the damaged telomeres. We also examined possible involvement of the HR pathway in the CPT-induced DSB repair. Since RAD51 is involved in the search for homology and strand pairing stages of the process [58, 59], we used confocal microscopy to examine the RAD51/TRF1 co-localization in CD4 T cells exposed to CPT or DMSO for 6 or 72 h. Again, we did not find any difference between control and treatment (Fig. 5d).

Since CPT induces cytotoxicity by trapping Top1cc at the Top 1-DNA covalent sites, which in turn interacts with advancing replication or transcription complexes to generate lethal DNA lesions, we further hypothesize that Top1cc may accumulate and occupy the DSB ends, preventing the recruitment of DNA repair molecules to the damaged sites in T cells. To test this possibility, we examined the CPT-induced, Top1cc-mediated dysfunctional telomere foci using a monoclonal antibody that specifically recognizes covalent Top 1-DNA complexes, but not free Top 1 or DNA, using confocal microscopy [28]. We found a significant increase in Top1cc/TRF1-formed TIFs in T cells exposed to CPT compared to the DMSO-treated control (Fig. 5e). Also, we observed Top1cc accumulation at damaged telomeres in T cells treated with KML001 (a telomere-targeting drug that can inhibit Top 1 expression and induce Top1cc), but not in cells treated with the ATM inhibitor (KU60019)-induced telomeric DNA damage, which is not involving in Top1cc induction but via the NHEJ pathway for DNA damage repair; unpublished observations). Taken together, these results suggest that CPT induces Top1cc accumulation and trapping at the DSB sites, which may prevent DNA repair molecule recruitment and inhibit DNA damage repair.

### T cells from individuals with chronic viral infection are more vulnerable to CPT-induced cell apoptosis

**Fig. 6:**
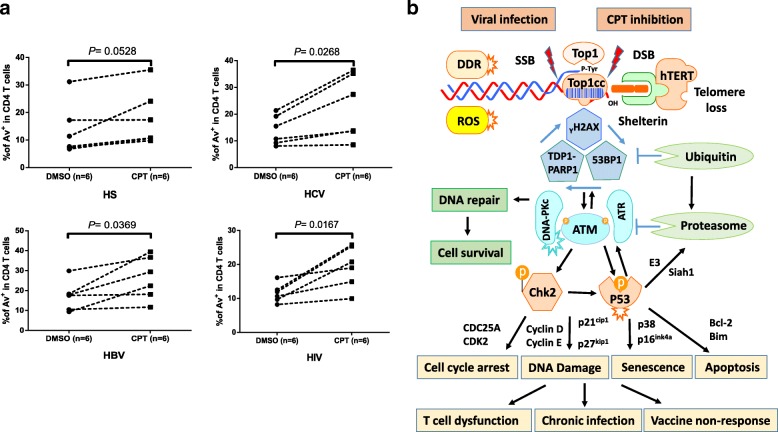
Sensitivity of CD4 T cells derived from virally infected individuals and HS to CPT-induced apoptosis, and a novel model of virus-induced disruption of DNA topology in T cell dysregulation. **a** Vulnerability of CD4 T cells, derived from virally infected individuals and HS, to the CPT-induced apoptosis as measured by flow cytometry. **b** A working model for Top 1-mediated DDR and T cell dysregulation during HIV infection. The intertwined nature of two complementary DNA often lead to topological entanglements during gene transcription and replication that must be resolved to ensure normal gene transactions and cell functions. In order to prevent and correct these types of topological problems, Top 1 binds to DNA and cuts one DNA strand, allowing the DNA to be untangled or unwound so as to exert genetic activities. According on our results, the immunomodulatory virus (HCV, HBV, HIV) infection and/or CPT treatment can inhibit Top 1 protein expression and enzymatic activity, leading to Top1cc trapped at the DNA breaks including telomere termini and causing topological DNA damage, telomere loss, cell senescence and apoptosis. This regulatory cascade represents a novel molecular mechanism that underlies T cell senescence and T cell dysfunction, which contribute to viral persistence and vaccine non-responsiveness in human viral infections

Since chronically virus-infected patients suffer from deficiency in the Top 1 enzyme and exhibit more DNA damage within their CD4 T cells, we hypothesized that these T cells were more vulnerable to CPT-mediated cell apoptosis. We thus examined the apoptotic susceptibility of CD4 T cells derived from virus-infected patients versus age-matched HS upon CPT treatment. Indeed, CD4 T cells isolated from HCV-, HBV-, and HIV-infected patients exhibited higher rates of cell apoptosis (Av expression) than those from HS (Fig. 6a). Correspondingly, a higher rate of cell death (7-AAD expression) was observed in CD4 T cells from virally infected patients versus HS (data not shown). These results, in conjunction with our previous in vivo findings of T cell exhaustion and senescence in virus-infected individuals [26, 27], suggest that Top1-mediated topological DNA damage, if left unrepaired, can trigger both cell apoptosis and cell death.

## Discussion

T cells play a pivotal role in controlling pathogenic infection and vaccine responses. In chronic viral infections, particularly in immunosuppressive HCV, HBV, and HIV infections; T cells are however essentially always dysfunctional and vaccine responses are often blunted [60]. We and others have previously shown that T cells from chronically virus-infected individuals are prematurely aged or senescent due to telomere attrition and erosion [1–13, 26, 27], but the underlying mechanisms for T cell telomeric DNA damage and repair remain unclear. Since Top 1 is required to remove DNA supercoiling generated during cell overexpansion, and TOP1cc can become trapped during gene transcription to cause Top 1-linked PDB [20–23], we hypothesized that T cell DNA topology may be altered during viral infections to cause DDR as a mechanism of virus-mediated immune dysregulation.

In this study, we employed T cells isolated from virus-infected individuals and primary T cells treated with CPT as a model to specifically induce transcription-blocking Top1cc in order to obtain insights into the molecular mechanisms underlying both DNA damage and repair processes. We demonstrate that: 1) CD4 T cells derived from chronically HBV, HCV, and HIV-infected individuals with immune dysfunction exhibit an inhibition of Top 1 enzyme expression and activity, leading to accumulation of Top1cc and DNA damage, including telomere erosion; 2) CPT-induced Top 1 inhibition, Top1cc accumulation, topological DNA damage, telomere uncapping and erosion, T cell apoptosis and dysfunctions recapitulate what we observe in T cells during chronic viral (HCV, HBV, HIV) infections, emphasizing the role of Top 1 in maintaining telomeric DNA integrity and securing T cell survival and function; 3) CPT-induced topological DDR is achieved via transcription-dependent, ubiquitin-mediated Top 1 proteolysis, involving dynamic ATM activation followed by deficiency - Top1cc accumulation at the DSB sites may block the recruitment of DNA repair proteins and inhibit DNA damage repair; and 4) T cells from virally infected subjects with lower Top 1 activity are more vulnerable to CPT-induced topological DNA damage and cell apoptosis, indicating an important role for Top 1 in preventing unwanted DNA damage and securing cell survival.

Although accumulation of DNA damage and failure to be repaired may affect cell survival and function, the molecular signaling pathways in T lymphocytes in the context of chronic viral infections remain largely unknown. Our results support a model, as depicted in Fig. 6b, in which Top 1 inhibition and Top1cc accumulation block transcription elongation, which triggers ubiquitin-mediated Top 1 proteolysis and the generation of Top1cc-linked PDBs. Defective repair of these PDBs can further give rise to DDR, which leads to ATM activation and phosphorylation of its downstream substrates such as CHK2 and p53. ATM also activates 53BP1 and γH2AX assembling into nuclear DNA damage foci and promotes ubiquitination of multiple signaling molecules in the process of DNA damage and repair. Notably, several E3 ligases have been reported for γH2AX (RNF2, RNF8, RNF168) and for Top 1 (Cullin3, Cullin4A/4B, BRCA1) ubiquitination and proteasomal degradation [45, 61]. Our findings that Top 1 inhibition and γH2AX production in CPT-treated T cells depend both on transcription and on Top 1 ubiquitination suggest that they arise during the repair of Top1cc and that this pathway feedback to enhance Top1cc repair after Top 1-linked PDB induction. Moreover, inhibition of PARP1, which is required for TDP1 excision of Top1cc [40–42], also increases transcription-dependent Top 1-PDB and γH2AX induction following Top1cc stabilization. This model is supported by our recent findings showing that ATM deficiency [26] and TRF2 uncapping [27] increases DNA damage, telomere attrition, T cell senescence and apoptosis, likely due to increased Top1cc levels, as demonstrated in this study in both T cells isolated from chronic viral infections or treated with CPT, leading to T cell dysfunction.

While the mechanisms that lead to Top 1 inhibition during chronic viral infections remain unclear, multiple factors may play a role in this process. Topological DNA damage might occur spontaneously in replicating T cells under physiological conditions, but Top1cc can be trapped under a broad range of pathological conditions, including Top 1 inhibition by immunomodulating viruses (HCV, HBV, HIV, and coinfection with CMV or EBV), by oxidative base damage, by alkylation with carcinogenic compounds or antiviral agents, or by ribonucleotide misincorporations during genetic activities in over-proliferating T cells in response to low grade, chronic inflammatory stimuli [20–25]. Therefore, these transcriptional Top 1-linked PDBs may have a marked impact on senescent T cells, leading to reprogramming DDR and remodeling of the T cell fate that arise from i) defective removal of Top1cc, ii) deprotection of telomeres by shelterins [27], iii) failure of telomere elongation by telomerase dysfunction, and iv) deficiency of DNA damage repair by ATM [26]. T cells might be particularly prone and vulnerable to Top 1-mediated topological DNA damage and cell apoptosis as a result of high rates of oxygen consumption, which produces large amounts of reactive oxygen species (ROS), which we have recently found to be significantly increased in T cells of virus- (HCV and HIV)-infected individuals. Indeed, ROS can stabilize Top1cc to cause topological DNA damage and activate the ATM signaling pathway [28, 29, 62, 63]. Hence, our findings suggest a new paradigm in which topological DNA damage contributes to the T cell senescence, apoptosis and immune evasion occurring during chronic viral infections.

This study focuses on the major blood-borne pathogens, HBV, HCV, and HIV. It would be noteworthy to investigate whether topological DNA damage and premature T cell aging also occur in the setting of other chronic viral infections, such as CMV and EBV. These infections often display similar phenotypic characteristics to those observed in HIV, HBV and/or HCV infection, such as exhausted and senescent T cells, and this would be especially relevant in immunocompromised hosts. Our investigation in CD4 T cells is informative because they represent the lymphocyte helper subsets of the immune system, aiding in CD8 T cell and B cell responses, and their survival critically affects the process of immune aging. We expect to see the same topological DNA damage in CD8 T cells, which often display more prominent dysfunctions with exhaustion and senescence phenotype. It should be pointed out that while Top 1 inhibition and Top1cc accumulation explain both telomeric DNA damage and cell apoptosis, they may function as a double-edged sword, resulting in both overwhelming cell death in acute infection and immune tolerance or immune suppression in chronic infection. Nevertheless, these findings provide the first demonstration of a pivotal role for Top 1 in DDR and new molecular insights into immunomodulation during chronic viral infections through disruption of Top 1 to induce telomeric DDR, T cell senescence, apoptosis, and dysfunction. Most importantly, it provides a potential strategy to restore impaired DNA topological machinery as a means to improve T cell functions and vaccine responses during human viral infections.

## Conclusion

We and others have previously shown that chronic viral infections could cause premature T cell aging or immune senescence with accelerated telomere erosion, but the mechanisms underlying DNA damage and telomere loss remain unclear. For the first time to our knowledge, here we report that Top 1 was inhibited, and TOP1cc was trapped in genomic DNA, in T cells from patients with chronic HBV, HCV, and HIV infection. We then used healthy T cells treated with CPT as a model to investigate the role and mechanisms of DNA topology in reprogramming telomeric DDR and T cell functions. We demonstrated that Top 1 inhibition caused topological DNA damage, telomere attrition, and T cell apoptosis or dysfunction by inducing Top1cc accumulation and failure in DNA repair, thus recapitulating T cell dysregulation in the setting of chronic viral infections. This study reveals a novel mechanism for immunomodulation by viral infections via disrupting DNA topology, telomere integrity, and T cell biology. Therefore, restoring the impaired DNA topologic machinery may offer a new strategy to maintain T cell function against human viral diseases.

## Data Availability

The data and material are available upon request.

## Abbreviations

ATM: Ataxia telangiectasia mutated
ATR: Ataxia telangiectasia Rad3-related
CPT: Camptothecin
DDR: DNA damage responses
DMSO: Dimethyl sulfoxide
DNA-PKc: DNA protein kinase c
DSB: Double strand break
DUSP6: Dual specific phosphatase 6
Flow-FISH: Florescence in-situ hybridization
HBV: Hepatitis B virus
HCV: Chronic hepatitis C virus
HIV: Human immunodeficiency virus
HR: Homologous recombination
HS: Healthy subjects
hTERT: Human telomerase reverse transcriptase
KLRG1: Killer cell lectin-like receptor superfamily G 1
miR: microRNA
NHEJ: Non-homologous end joining
PARP1: Poly ADP-Ribose Polymerase 1
PBMCs: Peripheral blood mononuclear cells
PCR: Polymerase chain reaction
PDB: Protein-linked DNA breaks
POT1: Protection of telomere 1
RAP1: Repressor/activator protein 1
TCR: T cell receptor
TDP1: Tyrosyl-DNA phosphodiesterase-1
TIN2: TRF1-interacting nuclear protein 2
Top 1: Topoisomerase I
TOP1cc: Top 1 cleavage complex
TPP1: Telomere protection protein 1
TR: Telomerase RNA
TRF1/2: Telomeric repeat-binding factor 1/2

## References

[1] Cao D, Zhao J, Nguyan LN, Nguyen LNT, Khanal S, Dang X, Schank M, Chand Thakuri BK, Wu XY, Morrison ZD, Ei Gazzar M, Zou Y, Ning S, Wang L, Moorman JP, Yao ZQ (2019). Disruption of Telomere Integrity and DNA Repair Machineries by KML001 Induces T Cell Senescence, Apoptosis, and Cellular Dysfunctions. Front Immunol..

[2] Shi L, Wang JM, Ren JP, Cheng YQ, Ying RS, Wu XY, Lin SM, Griffin JWD, Li GY, Moorman JP, Yao ZQ (2014). KLRG1 impairs CD4^+^ T cell responses via p16^ink4a^ and p27^kip1^ pathways: role in hepatitis B vaccine failure in individuals with hepatitis C virus infection. J Immunol.

[3] Li G, Zhou Y, Ying RS, Shi L, Cheng YQ, Ren JP, Griffin JWD, Jia ZS, Li CF, Moorman JP, Yao ZQ (2014). HCV induced reduction in miR-181a impairs CD4+ T cell responses via over-expression of DUSP6. Hepatology.

[4] Zhou Y, Li GY, Ren JP, Wang L, Zhao J, Ning SB, Zhang Lian JQ, Huang CX, Jia ZS, Moorman JP, Yao ZQ (2016). Protection of CD4^+^ T cells from HCV infection-associated senescence via ΔNp63-miR181a-Sirt1 pathway. J Leukc Bio.

[5] Hoare M, Gelson WT, Das A, Fletcher JM, Davies SE, Curran MD, Vowler SL, Maini MK, Akbar AN, Alexander GJ (2010). CD4+ T-lymphocyte telomere length is related to fibrosis stage, clinical outcome and treatment response in chronic hepatitis C virus infection. J Hepatol.

[6] Biron-Shental T, Amiel A, Anchidin R, Sharony R, Hadary R, Kitay-Cohen Y (2013). Telomere length and telomerase reverse transcriptase mRNA expression in patients with hepatitis C. Hepatogastroenterology..

[7] Barathan M, Mohamed R, Yong YK, Kannan M, Vadivelu J, Saeidi A, Larsson M, Shankar EM. Viral Persistence and Chronicity in Hepatitis C Virus Infection: Role of T-Cell Apoptosis, Senescence and Exhaustion. Cells. 2018;7(10). 10.3390/cells7100165 PMC6210370 30322028

[8] Cobos Jiménez V, Wit FW, Joerink M, Maurer I, Harskamp AM, Schouten J (2016). T-cell activation independently associates with immune senescence in HIV-infected recipients of long-term antiretroviral treatment. J Infect Dis.

[9] Zanet DL, Thorne A, Singer J, Maan EJ, Sattha B, Le Campion A (2014). Association between short leukocyte telomere length and HIV infection in a cohort study: no evidence of a relationship with antiretroviral therapy. Clin Infect Dis.

[10] Nelson JA, Krishnamurthy J, Menezes P, Liu Y, Hudgens MG, Sharpless NE, Eron JJ (2012). Expression of p16(INK4a) as a biomarker of T-cell aging in HIV-infected patients prior to and during antiretroviral therapy. Aging Cell.

[11] Grady BP, Nanlohy NM, van Baarle D (2016). HCV monoinfection and HIV/HCV coinfection enhance T-cell immune senescence in injecting drug users early during infection. Immun Ageing.

[12] Gross AM, Jaeger PA, Kreisberg JF, Licon K, Jepsen KL, Khosroheidari M (2016). Methylome-wide analysis of chronic HIV infection reveals five-year increase in biological age and epigenetic targeting of HLA. Mol Cell.

[13] Ferrando-Martínez S, Ruiz-Mateos E, Romero-Sánchez MC, Muñoz-Fernández MÁ, Viciana P, Genebat M, Leal M (2011). HIV infection-related premature Immunosenescence: high rates of immune exhaustion after short time infection. Curr HIV Res.

[14] Wong JM, Collins K (2003). Telomere maintenance and disease. Lancet.

[15] Carneiro MC, de Castro IP, Ferreira MG (2016). Telomeres in aging and disease: lessons from zebrafish. Dis Model Mech.

[16] Blackburn EH, Greider CW, Szostak JW (2006). Telomeres and telomerase: the path from maize, Tetrahymena and yeast to human cancer and aging. Nat Med.

[17] Arkus N (2005). A mathematical model of cellular apoptosis and senescence through the dynamics of telomere loss. J Theoretical Biol.

[18] Champoux JJ (2001). DNA topoisomerases: structure, function, and mechanism. Annu Rev Biochem.

[19] Wang JC (2002). Cellular role of DNA topoisomerases: a molecular perspective. Nature Rev Mol Cell Biol.

[20] Pommier Y (2006). Repair of topoisomerase I-mediated DNA damage. Nuclear Acid Res Mol Biol.

[21] Vos SM, Tretter EM, Schimidt BH, Berger JM (2011). All tangled up: how cells direct, manage and exploit topoisomerase function. Nature Rev Mol Cell Biol..

[22] Pouliot JJ, Yao KC, Robertson CA, Nash HA (1999). Yeast gene for a Tyr-DNA phosphodiesterase that repairs topoisomerase I complexes. Science.

[23] Guo D, Dexheimer TS, Pommier Y, Nash HA (2014). Neuroprotection and repair of 3′-blocking DNA ends by glaikit (gkt) encoding Drosophila tyrosyl-DNA phosphodiesterase 1 (TDP1). Proc Natl Acad Sci U S A.

[24] Pommier Y, Leo E, Zhang H, Marchand C (2010). DNA topoisomerases and their poisoning by anticancer and antibacterial drugs. Chem Biol.

[25] Pommier Y (2013). Drugging topoisomerases: lessons and challenges. ACS Chem.

[26] Zhao J, Dang X, Zhang P, Nguyen L, Cao D, Wang L, Wu X, Morrison Z, Zhang Y, Jia Z, Xie Q, Wang L, Ning S, EL Gazzar M, Moorman J, Yao ZQ (2018). Insufficiency of DNA repair enzyme ATM promotes naïve CD4 T cell loss in chronic hepatitis C virus infection. Cell Discov.

[27] Nguyen L, Zhao J, Cao D, Dang X, Lian J, Zhang Y, Jia Z, Wu X, Morrison Z, Wang L, Ning S, Xie Q, El Gazzar M, Zhang Z, Moorman J, Yao Z (2018). Inhibition of telomeric repeat binding factor 2 accelerates telomere attrition and DNA damage in naïve CD4 T cells during chronic HCV infection. Cell Death Dis.

[28] Patel AG, Flatten KS, Peterson KL, Beito TG, Schneider PA, Perkins AL, Harki DA, Haufmann SH (2016). Immunodetection of human topoisomerase I-DNA covalent complexes. Nucleic Acid Research.

[29] Pommier Y (2006). Topoisomerase I inhibitors: camptothecins and beyond. Nat Rev Cancer.

[30] Henle ES, Han Z, Tang N, Rai P, Luo Y, Linn S (1999). Sequence-specific DNA cleavage by Fe2+−mediated Fenton reactions has possible biological implications. J Biol Chem.

[31] Petersen S, Saretzki G, von Zglinicki T (1998). Preferential accumulation of single-stranded regions in telomeres of human fibroblasts. Exp Cell Res.

[32] Rothkamm K, Barnard S, Moquet J, Ellender M, Rana Z, Burdak-Rothkamm S (2015). DNA damage foci: meaning and significance. Environ Mol Mutagen.

[33] Takai H, Smogorzewska A, de Lange T (2003). DNA damage foci at dysfunctional telomeres. Curr Biol.

[34] Capranico G, Ferri F, Fogli MV, Russo A, Lotito L, Baranello L (2007). The effects of camptothecin on RNA polymerase II transcription: roles of DNA topoisomerase I. Biochimie..

[35] Ljungman M, Lane DP (2004). Transcription – guarding the genome by sensing DNA damage. Nat Rev Cancer.

[36] Desai SD, Zhang H, Rodriguez-Bauman A, Yang JM, Wu X, Gounder MK, Rubin EH, Liu LF (2003). Transcription-dependent degradation of topoisomerase I-DNA covalent complexes. Mol Cell Biol.

[37] El-Khamisy SF, Saifi GM, Weinfeld M, Johansson F, Helleday T, Lupski JR, Caldecott KW (2005). Defective DNA single-strand break repair in spinocerebellar ataxia with axonal neuropathy-1. Nature.

[38] Miao ZH, Agama K, Sordet O, Povirk L, Kohn KW, Pommier Y (2006). Hereditary ataxia SCAN1 cells are defective for the repair of transcription-dependent topoisomerase I cleavage complexes. DNA Rep.

[39] Ashour ME, Atteya R, El-Khamisy SF (2015). Topoisomerase-mediated chromosomal break repair: an emerging player in many games. Nat Rev Cancer.

[40] Pommier Y, Barcelo JM, Rao VA, Sorfet O, Jobson AG, Thibaut L, Maio ZH, Seiler JA, Zhang H, MArchand C (2006). Repair of topoisomerase I-mediated DNA damage. Prog Nucleic Acid Res Biol.

[41] Bouglares AH, Yakovlev G, Ivanova V (1999). Role of poly (ADP-ribose) polymerase (PARP) cleavage in apoptosis. Caspase 3-resistant PARP mutant increases rates of apoptosis in transfected cells. J Bio Chem.

[42] Das BB, Huang SY, Murai J, Rehman I, Ame JC, Sengupta S, Das SK, Majumdar P, Zhang H, Biard D (2014). PARP1-TDP1 coupling for the repair of topoisomerase I-induced DNA damage. Nucleic Acid Res.

[43] Somnay Y, Lubner S, Gill H, Matsumura JB, Chen H (2016). The PARP inhibitor ABT-888 potentiates darbazine-induced cell death in carcinoids. Cancer Gene Ther.

[44] Desai SD, Li TK, Rodriguez-Bauman A, Rubin EH, Liu LF (2001). Ubiquitin/26S proteasome-mediated degradation of topoisomerase I as a resistance mechanism to camptothecin in tumor cells. Cancer Res.

[45] Dantuma NP, Groothuis TA, Salomons FA, Neefjes J (2006). A dynamic ubiquitin equilibrium couples proteasomal activity to chromatin remodeling. J Cell Biol.

[46] Kalathiya U, Padariya M, Baginski M (2019). Structural, functional, and stability change predictions in human telomerase upon specific point mutations. Sci Rep.

[47] Blackburn EH (2000). Telomere states and cell fates. Nature.

[48] Akbar AN, Vukmanovic-Stejic M (2007). Telomerase in T lymphocytes: use it and lose it?. J Immunol.

[49] De Lange T (2005). Shelterin: the protein complex that shapes and safeguards human telomeres. Genes Dev.

[50] Giraud-Panis MJ, Pisano S, Benarroch-Popivker D, Pei B, Le Du MH, Gilson E (2013). One identity or more for telomers?. Front Oncol.

[51] Uziel T, Lerenthal Y, Moyal L, Andegeko Y, Mittelman L, Shiloh Y (2003). Requirement of the MRN complex for ATM activation by DNA damage. EMBO J.

[52] Dupre A, Boyer-Chatenet L, Gautier J (2006). Two-step activation of ATM by DNA and the Mre11-Rad50-Nbs1 complex. Nat Struct Mol Bio.

[53] Awasthi P, Foiani M, Kumar A (2015). ATM and ATR signaling at a glance. J Cell Sci.

[54] Bonner WM, Redon CE, Dickey JS, Nakamura AJ, Sedelnikova OA, Solier S, Pommier Y (2008). gammaH2AX and cancer. Nat Rev Cancer.

[55] Cristini A, Park JH, Capranico G, Legube G, Favre G, Sordet O (2016). DNA-PK triggers histone ubiquitination and signaling in response to DNA double-strand breaks produced during the repair of transcription-blocking topoisomerase I lesions. Nuclear Acids Res.

[56] Li H, Vogel H, Holcomb VB, Gu Y, Hasty P (2007). Deletion of Ku70, Ku80, or both causes early aging without substantially increased cancer. Mol Cell Biol.

[57] Boulton SJ, Jackson SP (1998). Components of the Ku-dependent non-homologous end-joining pathway are involved in telomeric length maintenance and telomeric silencing. EMBO.

[58] Shinohara A, Ogawa H, Ogawa T (1992). Rad51 protein involved in repair and recombination in S. cerevisiae is a RecA-like protein. Cell..

[59] Kawabata M, Kawabata T, Nishibori M (2005). Role of recA/RAD51 family proteins in mammals. Acta Med Okayama.

[60] Yao ZQ, Moorman JP (2013). Immune exhaustion and immune senescence: two distinct pathways for HBV vaccine failure during HCV and/or HIV infection. Arch Immunol Ther Exp.

[61] Pan MR, Peng G, Hung WC, Lin SY (2011). Monoubiquitination of H2AX protein regulates DNA damage response signaling. J Biol Chem.

[62] Daroui P, Desai SD, Li TK, Liu AA, Liu LF (2004). Hydrogen peroxide induces topoisomerase I-mediated DNA damage and cell death. J Biol Chem.

[63] Guo Z, Kozlov S, Lavin MF, Person MD, Paull TT (2010). ATM activation by oxidative stress. Science..

